# Priming with γ-Aminobutyric Acid against *Botrytis cinerea* Reshuffles Metabolism and Reactive Oxygen Species: Dissecting Signalling and Metabolism

**DOI:** 10.3390/antiox9121174

**Published:** 2020-11-25

**Authors:** Henry Christopher Janse van Rensburg, Wim Van den Ende

**Affiliations:** Laboratory of Molecular Plant Biology, KU Leuven, Kasteelpark Arenberg 31, 3001 Leuven, Belgium; henry.jansevanrensburg@kuleuven.be

**Keywords:** γ-aminobutyric acid (GABA), *Botrytis cinerea*, reactive oxygen species (ROS), sugars, priming, signalling

## Abstract

The stress-inducible non-proteinogenic amino acid γ-aminobutyric acid (GABA) is known to alleviate several (a)biotic stresses in plants. GABA forms an important link between carbon and nitrogen metabolism and has been proposed as a signalling molecule in plants. Here, we set out to establish GABA as a priming compound against *Botrytis cinerea* in *Arabidopsis thaliana* and how metabolism and reactive oxygen species (ROS) are influenced after GABA treatment and infection. We show that GABA already primes disease resistance at low concentrations (100 µM), comparable to the well-characterized priming agent β-Aminobutyric acid (BABA). Treatment with GABA reduced ROS burst in response to flg22 (bacterial peptide derived from flagellum) and oligogalacturonides (OGs). Plants treated with GABA showed reduced H_2_O_2_ accumulation after infection due to increased activity of catalase and guaiacol peroxidase. Contrary to 100 µM GABA treatments, 1 mM exogenous GABA induced endogenous GABA before and after infection. Strikingly, 1 mM GABA promoted total and active nitrate reductase activity whereas 100 µM inhibited active nitrate reductase. Sucrose accumulated after GABA treatment, whereas glucose and fructose only accumulated in treated plants after infection. We propose that extracellular GABA signalling and endogenous metabolism can be separated at low exogenous concentrations.

## 1. Introduction

The non-proteinogenic amino acid γ-aminobutyric acid (GABA) makes up a major part of the free amino acid pool and can be found in all living organisms. Besides its wide distribution over different kingdoms of life, the metabolic enzymes involved in GABA metabolism are also highly conserved among organisms [1]. Plants contain the highest level of GABA, specifically during periods of stress [2]. In plants, GABA is mainly synthesized from glutamate (Glu) through the cytosolic Glu decarboxylase (GAD) [3]. This reaction forms part of the GABA shunt, where GABA is subsequently catabolized to succinic semi-aldehyde (SSA) by GABA transaminase (GABA-T) followed by the oxidation of SSA by SSA dehydrogenase to produce succinate, which can directly feed into the tricarboxylic acid (TCA) cycle [4,5]. Alternatively, GABA can be produced by the oxidation of polyamines through polyamine oxidases [6]. It has also been proposed that under oxidative stress, proline can be oxidized non-enzymatically to produce GABA [7].

In animals, it is well known that GABA is closely associated with metabolic pathways and acts as an inhibitory neurotransmitter through GABA receptors [8]. In plants, much attention has been paid to the role of GABA during the regulation of plant developmental processes, such as pollen development [9], vasculature development [10], root growth [11], and *Lemna* rhizoid growth, with effects already being observed in the 1–10 µM range [12]. GABA also fulfils a central role in (a)biotic stress responses [13,14,15,16,17,18,19]. However, recent research shifted towards understanding the role of GABA as a signalling entity during several physiological processes, although it is still under debate [20,21,22,23,24]. In particular, it has been shown that GABA is central in the regulation of carbon:nitrogen (C:N) ratios because of its tight association with the TCA cycle [5]. GABA is particularly important during adaptation to environmental stresses, such as N starvation, salinity, drought, and low light [17,25,26,27]. During these conditions, it has been shown that GABA promotes growth and the antioxidant capacity of plants, suggesting a role for GABA in pro-survival strategies. Another indication that GABA is central in the outcome of cell death or survival is that it regulates the recycling and reallocation of N during leaf senescence [28]. GABA also performs key functions during defence against necrotrophic pathogens and insects [29,30,31,32].

The complex metabolic and signalling functions of GABA are believed to be intricately linked and intertwined in plants and are thus difficult to disentangle from each other [14]. In plants as autotrophic organisms, the two main “feeding” strategies are through the roots to obtain N and minerals, and through photosynthesis for C. Interestingly, GABA metabolism occurs sequentially in both the mitochondrial matrix and the cytosol and largely depends on environmental conditions. The ability of GABA to target mitochondrial metabolism to promote photosynthesis is well established [33,34]. The GABA shunt, for instance, is a major constituent for optimal photosynthesis in cyanobacteria [35]. Under hypoxia stress, exogenous GABA promoted photosynthesis in muskmelon plants [27]. In this regard, low exogenous GABA was proposed to act as a “stress” signal in plants. It is also believed that GABA act as a signal during the uptake of nutrients by the roots in a variety of plant species, including *Arabidopsis* [17] and *Brassica napus* [36]. Most of these observations appear to be concentration-dependent and strongly influenced by environmental conditions. Interestingly, most of these GABA-mediated effects on plants can be inhibited by antagonists or inhibitors of mammalian GABA receptors. This strongly suggests that conserved functions and potential receptors are shared between plants and animals [22]. It is therefore proposed that under conditions of low energy or starvation, GABA accumulates to promote the supply of energy and nutrients, very similar to the situation in animals. Sugars are crucial metabolites and signalling entities in plants [37,38,39], linking to the major energy sensors sucrose non-fermenting related kinase-1 (SnRK1) and target of rapamycin (TOR). Although there is no clear direct link established between GABA and these energy sensors, indirect connections between SnRK1/TOR and GABA signalling may be into place through 14-3-3 protein action. Indeed, exogenous GABA represses expression of 14-3-3 genes, which are known regulators of key C and N metabolic processes [15] and 14-3-3 proteins interact with the SnRK1 signalling complex to control nitrate reductase (NR) activity [40].

During stress, GABA originates from the breakdown of Glu through GAD, which is activated by an increase in cytosolic Ca^2+^ [21]. Pathogen-induced accumulation of Ca^2+^ in the cytosol is thus closely associated with GAD activity and GABA accumulation [41,42]. GABA accumulation is typically associated with increased tolerance against necrotrophic pathogens, and exogenous GABA elicits similar responses [30,32]. Similar to its effect during abiotic stress, GABA promotes pro-survival processes in the cells directly surrounding the necrotic lesion by constantly replenishing the TCA cycle through the GABA shunt [30]. Such pro-survival strategies can be particularly effective against necrotrophic pathogens favouring cell death. Exogenous treatment of GABA can thus be effective against necrotrophic pathogens like *B. cinerea* in the leaves and fruits of plants [30,43]. Besides acting directly as a C and N source, exogenous GABA also promotes N metabolism, including its own synthesis, under stress conditions [17,43].

A striking link exists between GABA and reactive oxygen species (ROS) homeostasis in plants. The GABA shunt effectively bypasses two enzymes of the TCA cycle that are sensitive to oxidative stress [5]. Thus, plants with a deficient GABA shunt are more prone to ROS overaccumulation under stress, leading to necrosis and the hypersensitive response (HR). Later studies revealed that Ca^2+^-mediated activation of the GABA shunt under UV stress was responsible for ROS detoxification [44]. Several studies demonstrated that exogenous GABA alleviates abiotic stress in part by the activation of ROS scavenging enzymes and the ascorbate-glutathione cycle [26,45,46]. It was also shown that exogenous GABA was able to differentially control the expression of a NADPH-oxidase (responsible for the ROS burst) in the roots of *Caragana intermedia* plants during salt stress [47]. Intriguingly, lower concentrations (250 µM) induced expression, whereas higher concentrations (10 mM) had the opposite effect, suggesting that GABA metabolism and signalling can be separated in some cases [25,47]. Under biotic stress, GABA can also modulate ROS scavenging enzymes to control ROS homeostasis in tomato and pear fruits [18,32]. This is further supported by the fact that GABA limits the (HR) in necrotic lesions to prevent the spread of necrotrophs [30,48]. Thus, it can be hypothesized that the coregulation of GABA and ROS may control necrotrophic growth by adjusting metabolism and ROS dynamics in key areas of the developing lesions [30,49].

Altogether, GABA clearly forms an integral part of the response to abiotic and biotic stresses in plants by regulating key metabolic and stress response pathways. Emerging evidence points to a dual role for GABA, both acting as a metabolite and signalling entity. The aim of this study was to further explore this duality during GABA priming against *B. cinerea* in *Arabidopsis*, discriminating between GABA signalling and metabolic effects. The effect of GABA pre-treatment on elicitor-induced ROS burst was also studied. We further investigated the concentration-dependent effects of exogenous GABA on sugar and nitrogen metabolism in response to *B. cinerea* infection, and how this correlates with endogenous GABA levels. We discuss our results in favour of low exogenous GABA acting as a stress signal and high exogenous GABA as a non-stress signal.

## 2. Materials and Methods

### 2.1. Plant Material and Growth Conditions

*Arabidopsis thaliana* plants of the Col-0 ecotype were used in all experiments. Seeds were dispensed onto square pots (9 × 9 × 8 cm) containing potting soil and vermiculite in a 3:2 ratio so that each pot contained 5 plants. Plants were grown in a Conviron^®^ (Berlin, Germany) growth chamber under cool white fluorescent lamps of 100 μmol photons m^−2^ s^−1^ with a light cycle of 12 h light (21 °C) and 12 h dark (18 °C).

### 2.2. Priming Treatments

*Arabidopsis* (4–5 weeks old) soil-grown plants were used for priming experiments as explained [50]. Priming was conducted by spraying the leaves of plants with GABA (Sigma-Aldrich, St. Louis, MO, USA) or β-Aminobutyric acid (BABA) (Sigma-Aldrich, St. Louis, MO, USA) at 100 µM, 1 mM, and control (ddH_2_O containing 0.0001% Tween-20) treatment using a spraying bottle. Each pot was gently sprayed with 5 mL of solution at a distance of +/− 15 cm until the surface of the plant was completely covered. All priming agents were prepared in sterile ddH_2_O containing 0.0001% Tween-20 (Acros Organics, Morris Plains, NJ, USA) as surfactant.

### 2.3. B. cinerea Cultivation and Infection

In all experiments, the *B. cinerea* B05.10 strain was used [50,51]. To obtain fungal spores for infection, *B. cinerea* was cultivated on 24 g/L potato dextrose agar (PDA) for 14 days in the dark at 21 °C prior to harvesting. Spores were harvested using ddH_2_O containing 0.0001% Tween-20. The harvested spores were then filtered through sterilized glass wool to remove any residual hyphae. Infection buffer was prepared by diluting the spores to 1 × 10^5^ spores per mL in sterile 12 g/L potato dextrose medium. Infection control (IC) solution contained potato dextrose without spores. Spores were incubated in the infection buffer for 4 h before infection to allow synchronous germination.

Infections were performed as described [50]. Three source leaves per plant (rosette leaves 5–7) were cut, rinsed in ddH_2_O, and dried by blotting with paper towel. Leaves were placed with the adaxial side upwards in a square petri dish (Greiner Bio-One, Frickenhausen, Germany) lined with moist paper. The leaves were infected individually by adding 5 µL of infection buffer or IC buffer to the tip of each leaf using a pipette. Plates were sealed with parafilm and placed in an infection room with a 12-h day and 12-h night cycle and maintained at 18 °C. A representative image of lesions after 72 h of infection can be found in the Supplementary Materials (Figure S1). For disease progression analysis, photos were taken 72 h after infection and the lesion area determined using a reference and the software ImageJ 1.5T (https://imagej.nih.gov/ij/).

### 2.4. Elicitor-Induced ROS Burst Assays

To quantify the ROS burst, a method relying on the oxidation of L-012 was used [50,52]. Leaf disks were punched from source leaves using a 0.35 mm cork-borer and transferred to the wells of a 96-well flat-bottom white luminescence plate (Greiner Bio-One, Frickenhausen, Germany) containing 150 µL of sterile ddH_2_O. The plate was covered in aluminium foil and incubated in the growth chamber for 12–16 h for recovery. Subsequently, water was replaced with 100 µL of incubation solution (20 µM L-012 (Wako Chemicals, Osaka, Japan), and 1 µg/mL horseradish peroxidase (PanReac AppliChem, Darmstadt, Germany) prepared in ddH_2_O). Baseline ROS production was determined by measuring the luminescence for 30 min using a GloMax^®^-Multi Detection System (Promega GmbH, Mannheim, Germany). Subsequently, 100 µL of assay solution (incubation solution with elicitor) were added to all the wells and luminescence measured for 60 min at 2-min intervals with an integration time of 0.5 s. Elicitors were added to final concentrations of 100 nM for flg22 [52] and 0.2 mg/mL for OGs [53]. OGs were prepared as previously described [54]. Background readings were subtracted to obtain the elicitor-induced ROS only. Luminescence values were obtained as relative light-emitting units (RLU).

### 2.5. H_2_O_2_ Extraction and Quantification

The extraction and quantification of H_2_O_2_ was carried out by using the eFOX method [55]. Samples were flash frozen in liquid nitrogen, and extraction occurred immediately to prevent the loss of H_2_O_2_. Frozen plant material was ground in 20 volumes of ice-cold 5% trichloroacetic acid (TCA) using a mortar and pestle. Extracts were centrifuged at 15,000× *g* at 4 °C for 10 min, and 500 µL of extract added to 500 µL of eFOX reagent (500 µM ferrous ammonium sulphate (Honeywell-Fluka, Morris Plains, NJ, USA), 200 mM sorbitol, 200 µM xylenol orange (Honeywell-Fluka, Morris Plains, NJ, USA), and 1% EtOH prepared in 50 mM H_2_SO_4_). Reactions were incubated for 30 min at room temperature and the OD measured at 550 and 800 nm using a spectrophotometer (Spectronic Genesys 5, Thermo Scientific, Waltham, MA, USA). The concentration of H_2_O_2_ was calculated using a standard curve prepared from 30 % H_2_O_2_ (Sigma-Aldrich, St. Louis, MO, USA) in a range between 0 and 200 µM.

### 2.6. Antioxidant Enzyme Extraction and Activity Measurements

Antioxidant enzymes were extracted as previously described [50]. *Arabidopsis* leaves were ground in liquid nitrogen using a mortar and pestle. In total, 100 mg of material were combined with 300 µL of extraction buffer (100 mM phosphate buffer (pH 7.0), 0.1% Triton X-100, 15% glycerol, 1 mM PMSF, 1 mM ascorbic acid, and 0.35 mM β-mercaptoethanol) and enzymes extracted by grinding with a plastic micro pestle inside a 1.5-mL Eppendorf tube for 30 s. Extracts were then centrifuged for 15 min at 10,000× *g* at 4 °C. Enzymes were kept at −80 °C until use, except for APX, which was measured immediately.

Catalase (CAT) activity was measured as described previously [56,57]. Enzyme extract (40 µL) was added to assay buffer (2 mL 100 mM phosphate buffer (pH 7.0)) in a quartz glass cuvette. Background was measured for 30 s before the addition of 40 µL of 1 M H_2_O_2_ to start the reaction, and the decrease in OD_240_ measured for 5 min at 10 s intervals on a Spectronic Genesys 5 spectrophotometer (Thermo Scientific, Waltham, MA, USA). Areas of linear breakdown of H_2_O_2_ were considered to determine the activity. Blanks consisted of a general control (buffer and H_2_O_2_) and sample control (buffer and enzyme extract).

Ascorbate peroxidase (APX) activity was assayed as described [58,59]. Reactions were carried out in a total volume of 2 mL and consisted of 1.870 mL of 100 mM phosphate buffer (pH 7.0) containing 0.5 mM ascorbic acid and 30 µL of enzyme extract. Background was measured for 1 min before the reaction was started by the addition of 100 µL of 27 mM H_2_O_2_. The oxidation of ascorbic acid was measured as the decrease in OD at 290 nm for 5 min at 10 s intervals. A second blank consisted of reaction mixture without the addition of enzyme. Activity was determined in the linear range.

Guaiacol peroxidase (GPX) activity was measured using a colorimetric method with guaiacol as substrate [60]. Previously extracted enzyme (25 µL) was added to a reaction mixture consisting of 1.875 mL of 100 mM phosphate buffer (pH 7.0) and 1 mL of 25 mM guaiacol in a glass quartz cuvette. Background was measured for 1 min before the addition of 100 µL of 2 % H_2_O_2_ to initiate the reaction. The increase in absorbance at OD_480_ was measured for 5 min at 10 s intervals. The second blank contained no enzyme.

For all enzyme reactions, the activity was calculated from the linear range of the reaction and enzyme activity is expressed as units (U)·mg protein^−1^, where 1 U is equal to the change in OD of 0.01 per min.

### 2.7. Soluble Sugar Extraction, Processing, and Analysis

Soluble sugars were extracted and quantified as before [61]. Ground leaf material (100 mg) was extracted in 1 mL of ddH_2_O by boiling for 15 min at 95 °C. Samples were vortexed and centrifuged for 10 min at 15,000× *g* and 200 µL of supernatant applied to a Dowex^®^ (Acros Organics, Morris Plains, NJ, USA) anion and cation exchange column. Columns were subsequently rinsed 6 times with 200 µL of ddH_2_O. The flow through was diluted 1:1 in 20 µM rhamnose H_2_O as the internal standard. Samples were analysed by HPAEC-IPAD Dionex 5000 (Thermo Scientific, Waltham, MA, USA) with separation on a CarboPac^TM^ PA100 column (Thermo Scientific) and a mobile phase of 90 mM NaOH as described [50]. Concentrations were calculated using standards of 10 µM of each sugar run alongside.

### 2.8. Amino Acid Extraction and Quantification

Amino acids were extracted from 100 mg of ground leaf material by adding 1 mL of ddH_2_O and boiling for 15 min. Samples were centrifuged for 10 min at 15,000× *g* and supernatantly diluted (1:1) with 50 µM Nor-Valine (used as internal standard). Amino acids were analysed by reverse-phase HPLC (Shimadzu, Kyoto, Japan) by derivatization using o-Phtalaldehyde before injection. Separation was carried out using a YMC Triart C18 (YMC, Kyota, Japan) column and mobile phases consisting of buffer A (50 mM KH_2_PO_4_ pH 6.5, 0.7% *v*/*v* Tetrahydrofurane) and buffer B (Acetonitrile:MeOH:water 45:40:15) using the following gradient: 96% A and 4% B from 0 to 6 min; 92% A and 8% B from 6 to 18 min; 85% A and 15% B from 18 to 32 min; 67% A and 33% B from 32 to 50 min; 100% B from 50 to 53 min. Amino acids were detected using a fluorescence detector at λ_ex_ = 230 nm and λ_em_ = 450 nm.

### 2.9. Enzyme Extraction and Nitrate Reductase Activity Measurement

Nitrate reductase (NR) activity was measured as described previously [62]. Leaf samples were ground in liquid nitrogen, and 100 mg of fresh weight was extracted with 400 µL of extraction buffer (50 mM HEPES-KOH pH 7.5, 10% (*v*/*v*) glycerol, 0.1% Triton X-100, 10 mM MgCl_2_, 1 mM EDTA, 1 mM benzamidine, 1 mM ε-aminocapronic acid, 1 mM PMSF, 1 × cOmplete^™^ Protease Inhibitor Cocktail (Roche, Diagnostics Ltd., Mannheim, Germany), 1 mM DTT, and 20 µM flavin adenine dinucleotide). Samples were centrifuged at 13,000× *g* (4 °C) for 5 min and the supernatant used in reactions. Assays were performed by adding 100 µL of extract to 450 µL of assay buffer. Assay buffers consisted of 50 mM HEPES-KOH pH 7.5, 0.04% Triton X-100, 10 µM Na_2_MoO_4_, 0.5 mM DTT, and 20 mM KNO_3_, with the addition of 10 mM MgCl_2_ for the selective NR (active) reaction and 5 mM EDTA for the total NR reaction. The reaction mixture was incubated for 2 min at 25 °C before 50 µL of 3 mM NADH was added to start the reactions. Reactions were stopped by adding 50 µL of 0.6 mM zinc acetate. Blanks for both selective and total NR reactions were included by the addition of zinc acetate before adding enzyme. Excess NADH was removed by adding 75 µL of phenyl methosulphate to the reactions and by incubating for 15 min in the dark. Finally, 300 µL of 1% sulphanilamide in 3 N HCl and 300 µL of 0.02 % *N*-(1-napthyl)-ethylenediamine were added and incubated for 20 min for colour development. Samples were centrifuged at 14,000× *g* for 5 min and the supernatant used to measure the azo-dye formed at an OD of 540 nm using a Multiskan Ascent 96/384 Plate Reader (Thermo Scientific, Waltham, MA, USA). The concentration of nitrite formed per reaction was interpolated from a standard curve prepared between 0 and 200 µM using NaNO_2_.

### 2.10. Experimental Setup

The experimental setup is depicted in Figure S2. Plants were treated at 0 h by spraying with different treatments, followed by infection with *B. cinerea* 72 h after treatment. Disease progression was analysed 72 h after infection (144 h after treatment). Samples were harvested at 3, 24, and 72 h (time of infection) after treatment and 24 h after infection (96 h after treatment). Elicitor-induced ROS burst experiments were carried out 24 h after treatment. All samples were obtained from the source leaves (rosette leaves 5–7) of plants between 4 and 5 weeks old. Examples of the lesion developed after 72 h of infection can be found in Figure S1 of the Supplementary Materials.

### 2.11. Graphical Preparation and Statistical Analysis

Graphs were prepared using GraphPad Prism 8.0.0 and Inkscape 1.0 (https://inkscape.Org). Statistical analysis was performed using GraphPad Prism version 8.0.0 for Windows, GraphPad Software, San Diego, CA, USA, www.graphpad.com.

## 3. Results

### 3.1. GABA Primes Longer-Term Resistance against B. cinerea in Arabidopsis

To establish whether exogenous GABA can prime longer-term resistance against subsequent infection with *B. cinerea*, *Arabidopsis* WT (Col-0) plants were sprayed with either 100 µM or 1 mM GABA and compared to a positive control β-aminobutyric acid (BABA) at the same concentration. A period extending up to 72 h between treatments and infection was considered to allow for both signalling and metabolic effects (see Figure S2 for details). Treatments were compared to untreated and H_2_O-treated controls. Figure S1 shows representative pictures of the lesions after 72 h.

**Figure 1:**
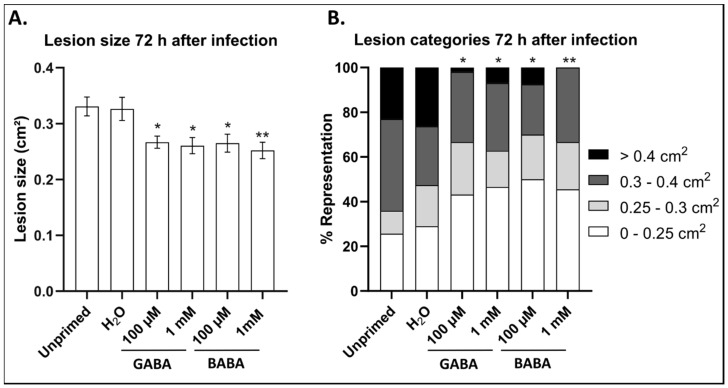
(**A**) Average lesion size of *Arabidopsis* plants infected with *B. cinerea* after treatment with 100 µM or 1 mM of either GABA or BABA compared to untreated and H_2_O controls. Bars represent the mean ± SE of at least 30 biological replicates. (**B**) Lesions of *B. cinerea* infection categorised according to size in plants pre-treated with either GABA or BABA compared to untreated and H_2_O control. Categories represent the distribution of at least 30 biological replicates. Asterisks indicate statistical significance compared to H_2_O according to one-way ANOVA followed by Dunnett’s multiple comparison test (* *p* < 0.05, ** *p* < 0.01). Experiments were repeated 3 times with consistent results.

Considering the average lesion size at 72 h after infection, plants pre-treated with both 100 µM and 1 mM GABA showed a significant reduction in susceptibility towards *B. cinerea* (Figure 1A). More than 60% of the lesions in plants treated with GABA were smaller than 0.3 cm^2^ whereas in the H_2_O control, more than 50% of the lesions were categorized larger than 0.3 cm^2^ (Figure 1B). It is also notable that less than 10% of the lesions in plants treated with GABA were larger than 0.5 cm^2^ whereas more than 20% of the lesions in the controls fell in this category. Plants treated with GABA showed comparable protective effects to those treated with BABA. Considering the most severe lesion category, GABA at 100 µM was more effective than BABA at the same concentration (Figure 1B). Thus, our data indicate that GABA can be equally effective as BABA, a well-established priming agent, and that low concentrations are sufficient to elicit such a response.

### 3.2. Exogenous GABA Negatively Regulates Elicitor-Mediated ROS Burst

An early defence signature of elicitor-induced responses in plants is the oxidative burst, also known as the ROS burst [63]. Elicitors, such as the bacterial peptide flg22, derived from flagellum, or OGs, derived from plant pectin fragments released upon fungal infection, trigger downstream signalling pathways to induce immune responses [63,64,65]. Priming agents like BABA, on the other hand, do not elicit these early defence responses but are known to prime defences, such as the ROS burst produced by NADPH-oxidases upon the perception of pathogens or elicitors [66]. On the other hand, GABA treatment during salt stress reduced the expression of an NADPH-oxidase in the roots of *Caragana intermedia* to confer tolerance [47]. Based on these studies, and the close association of GABA with ROS dynamics, we analysed the effect of exogenous GABA on NADPH-oxidase activity in response to flg22 and OGs.

**Figure 2:**
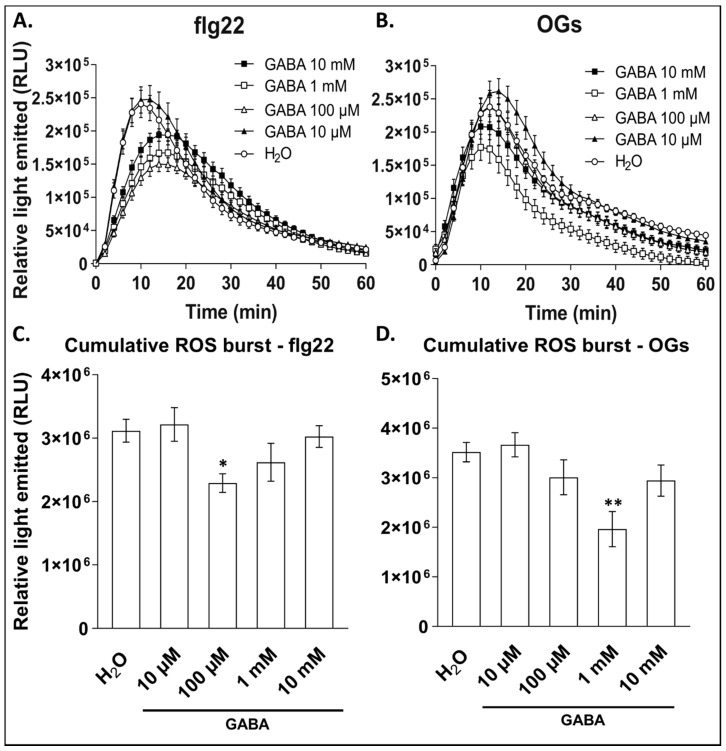
The effect of GABA pre-treatment at different concentrations on elicitor-induced ROS production in *Arabidopsis* leaf disks. Production of ROS in the leaf disks from plants pre-treated with different concentrations of GABA compared to H_2_O control in response to (**A**) 100 nM flg22 or (**B**) 0.2 mg/mL OGs. Each timepoint represents the mean ± SE (*n* = 16) and are expressed in relative light units (RLU). The cumulative ROS production over 60 min in response to (**C**) flg22 and (**D**) OGs after pre-treatment with H_2_O control or different concentrations of GABA. Bars represent the mean ± SE (*n* = 16) of the total ROS produced over 60 min. Statistical significance is indicated by an asterisk according to one-way ANOVA followed by Dunnett’s multiple comparisons test and adjusted *p* values (* *p* < 0.05; ** *p* < 0.01).

To establish the effect of exogenous GABA on the elicitor-induced ROS burst, plants were treated with a range of GABA concentrations as explained for infection studies, whereafter leaf disks from the source leaves were harvested 6 h later. After a recovery phase of 16 h, the leaf disks were treated with either flg22 or OGs while measuring the ROS burst for 60 min. We confirmed that the ROS burst profile in response to H_2_O pre-treatment did not significantly differ from untreated plants (Figure S3), and thus only the H_2_O control is shown. There is also no direct effect by GABA on the ROS burst, suggesting that GABA acts as a priming compound rather than as an elicitor (Figure S4). Plants pre-treated with GABA showed a reduction in ROS burst in response to both flg22 and OGs (Figure 2A,C). The ROS burst in plants pre-treated with 100 µM and 1 mM were the most affected in response to flg22 compared to the control. In response to OGs, a similar trend was observed, with the highest effect found in plants pre-treated with 1 mM GABA (Figure 2B,D). Interestingly, there seems to be a concentration limit for the inhibition of NADPH-oxidases by GABA, as plants pre-treated with 10 mM were less affected compared to 100 µM and 1 mM. Considering the cumulative ROS produced over a 60-min time window, the flg22-induced ROS burst was significantly reduced (>25%) by pre-treatment with 100 µM GABA compared to the control (Figure 2C). Pre-treatment with 1 mM also showed a lower cumulative ROS burst (>15% reduction) whereas at 10 µM and 10 mM, the effects were less prominent. Similarly, in response to OGs, plants pre-treated with 1 mM GABA showed a reduction of more than 40% compared to the control (Figure 2D). During treatments with 100 µM and 10 mM, the cumulative ROS burst was reduced by more than 20% compared to the control. In general, in response to OGs, the effect of GABA pre-treatment was more severe than for flg22.

### 3.3. Exogenous GABA Stimulates ROS Scavenging Enzymes during B. cinerea Infection

With a low concentration of exogenous GABA being able to induce immunity against *B. cinerea* and affecting elicitor-induced ROS production, the direct effect of GABA on H_2_O_2_ levels and H_2_O_2_ scavenging enzymes in the leaves of *Arabidopsis* plants were studied. To distinguish between the effect of leaf defoliation and infection, IC without spores were included alongside infected plants.

**Figure 3:**
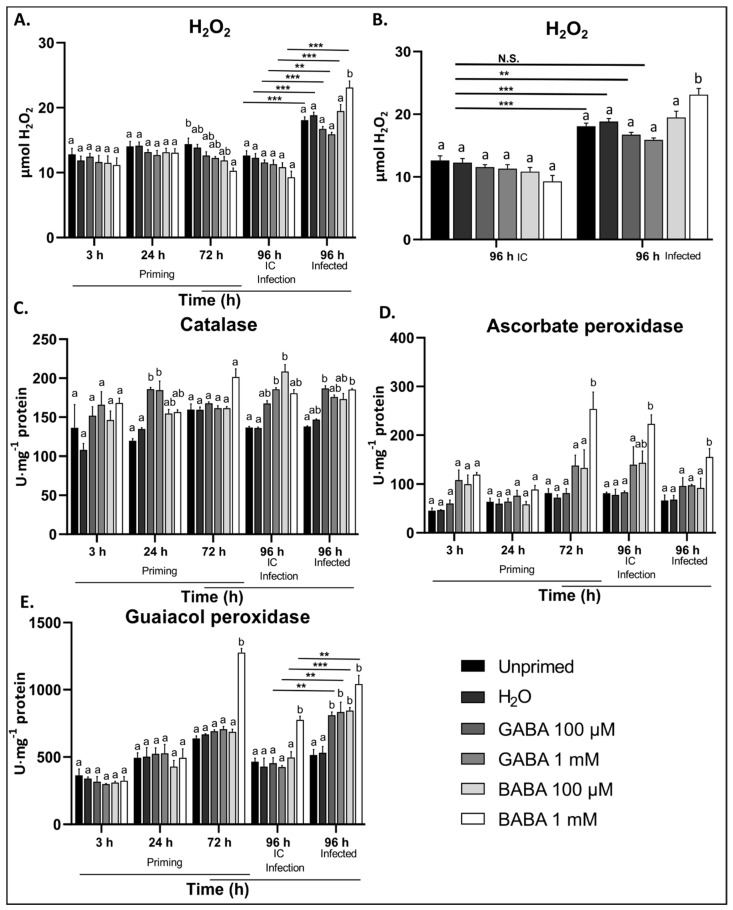
The effect of exogenous GABA on the level of H_2_O_2_ and H_2_O_2_ scavenging enzymes in the leaves of *Arabidopsis* plants infected with *B. cinerea*. Changes in H_2_O_2_ content (**A**,**B**) and the activities of (**C**) catalase, (**D**) ascorbate peroxidase, and (**E**) guaiacol peroxidase in response to GABA treatment and subsequent infection with *B. cinerea* compared to the positive control BABA and untreated and H_2_O-treated negative controls. Bars are the mean ± SE of 6 biological replicates for H_2_O_2_ and 3 biological replicates for catalase, ascorbate peroxidase, and guaiacol peroxidase. Different letters (a and b) indicate statistical significance within a timepoint (*p* < 0.05), and lines with asterisks between different timepoints and is based on two-way ANOVA and Tukey’s multiple comparison test and adjusted *p* values (not significant, N.S.; ** *p* < 0.01; *** *p* < 0.001). Experiments were repeated three times with consistent results. IC: infection control.

The H_2_O_2_ levels were not significantly affected by GABA pre-treatment at either concentration, while BABA treatments showed significantly reduced H_2_O_2_ levels 72 h after treatment (Figure 3A). The H_2_O_2_ content significantly increased after infection, independent of the treatment (Figure 3A). Plants pre-treated with 1 mM BABA showed significantly higher accumulation of H_2_O_2_ compared to the controls in response to *B. cinerea*, whereas GABA pre-treatment did not show significant differences. However, when comparing the changes in H_2_O_2_ as a consequence of infection, plants pre-treated with 1 mM GABA did not accumulate significantly higher levels compared to the IC controls (Figure 3E). This indicates that GABA pre-treatment does in fact significantly reduce the accumulation of H_2_O_2_ after *B. cinerea* infection when considering either controls as the basal H_2_O_2_ level. These data correlate well with the ROS burst experiments in response to elicitors, suggesting that GABA not only reduces the ROS produced in response to elicitors but also H_2_O_2_ as a consequence of *B. cinerea* infection. BABA treatment, on the other hand, showed higher H_2_O_2_ accumulation after infection.

In response to 100 µM and 1 mM GABA pre-treatment, only the activity of CAT was significantly higher at 24 h after treatment compared to the controls (Figure 3C), whereas APX did show temporal increases (not statistically significant) at 3 and 72 h after treatment with 1 mM GABA (Figure 3D). On the other hand, 1 mM BABA directly induced the activities of CAT, APX, and GPX at 72 h after treatment (Figure 3C–E). After leaf defoliation, CAT activity was significantly higher in plants pre-treated with GABA and BABA compared to controls (Figure 3C). Interestingly, GABA pre-treatment significantly primed the activities of CAT and GPX in response to *B. cinerea* infection, whereas BABA pre-treatment induced the activities already before infection. This suggests that low concentrations of GABA directly activate CAT activity but also prime the activities of CAT and GPX for subsequent stresses.

### 3.4. Exogenous GABA Leads to Increased GABA Levels in the Leaves

Through its ability to directly fuel the TCA cycle and bypass two steps sensitive to oxidative stress, GABA is an important component of the TCA cycle during *B. cinerea* infection [30]. As such, it is important to establish whether exogenous application of GABA influences endogenous GABA levels. Thus, we studied the time-dependent changes in both GABA and Glu (precursor of GABA) levels after GABA pre-treatment and *B. cinerea* infection.

**Figure 4:**
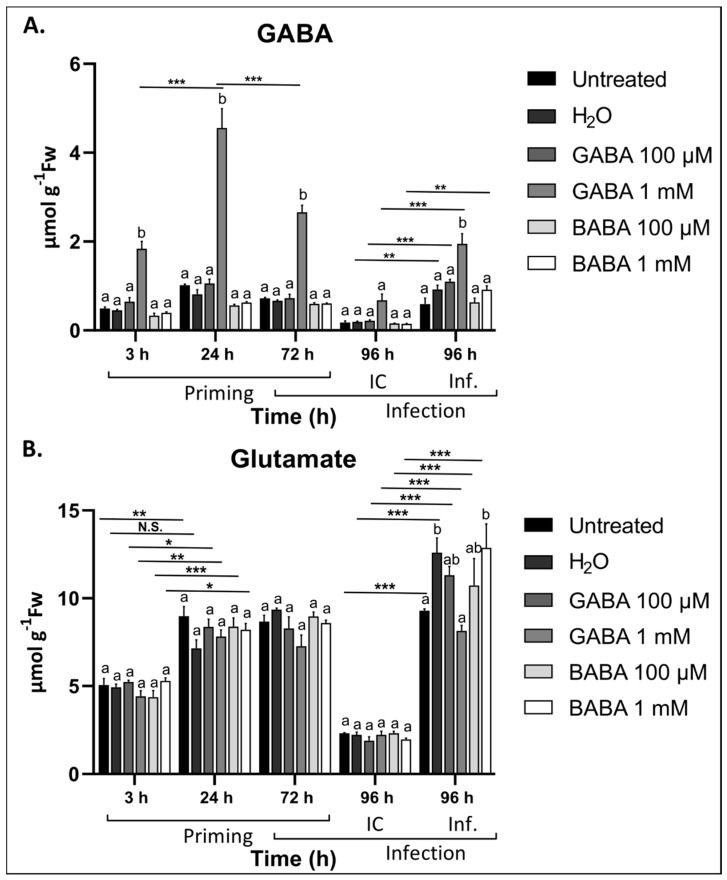
Changes in the total leaf GABA and Glu content in *Arabidopsis* after exogenous GABA treatment and infection with *B. cinerea*. Levels of (**A**) GABA and (**B**) Glu in *Arabidopsis* leaves after treatment with either 100 µM or 1 mM GABA followed by *B. cinerea* infection compared to untreated and H_2_O controls. Bars are the mean ± SE of 3 biological replicates. Significance is indicated by different letters (a and b) (*p* < 0.05) within the same timepoint, and with an asterisk between timepoints according to two-way ANOVA and Tukey’s multiple comparison test and adjusted *p* values (not significant, N.S.; * *p* < 0.05; ** *p* < 0.01; *** *p* < 0.001). IC: infection control. Inf: Infection.

Leaves sprayed with 1 mM GABA showed significantly higher levels of GABA, reaching a peak at 24 h after treatment (Figure 4A). Treatments with 100 µM GABA did not have any effect on the leaf GABA content. The level of Glu did not change significantly in response to either of the GABA treatments (Figure 4B). Leaf defoliation caused a reduction in both GABA and Glu levels, irrespective of the treatment (Figure 4A,B). In general, after infection with *B. cinerea*, both GABA and Glu levels were significantly higher in the leaves of infected plants compared to the IC. Interestingly, the GABA levels did not significantly change in response to infection in untreated plants, suggesting that even H_2_O treatment primes GABA accumulation after infection. This is interesting as the H_2_O control plants were also less susceptible to *B. cinerea* infection compared to untreated plants (Figure 1). Plants pre-treated with either 100 µM or 1 mM GABA showed the highest accumulation of GABA after infection with *B. cinerea*, suggesting that GABA might prime its own production after exposure to a pathogen. The level of Glu was significantly lower in plants treated with 1 mM GABA after infection when compared to the H_2_O control, indicating that Glu serves as a precursor for GABA production. On the other hand, treatments with BABA had no effect on GABA or Glu levels.

### 3.5. GABA Differentially Regulates Nitrate Reductase Activity

Previous studies showed that GABA can promote N uptake and utilization in *Arabidopsis* roots [17]. The effect of GABA on N metabolism in the leaves is less clear. However, since GABA can regulate the expression of 14-3-3 genes, which are direct regulators of NR activity, it seems possible that GABA can indirectly regulate the activity of NR in the leaves. To establish whether GABA regulates NR activity, we studied both the total and the active NR activity in response to exogenous GABA pre-treatment and subsequent infection with *B. cinerea*.

**Figure 5:**
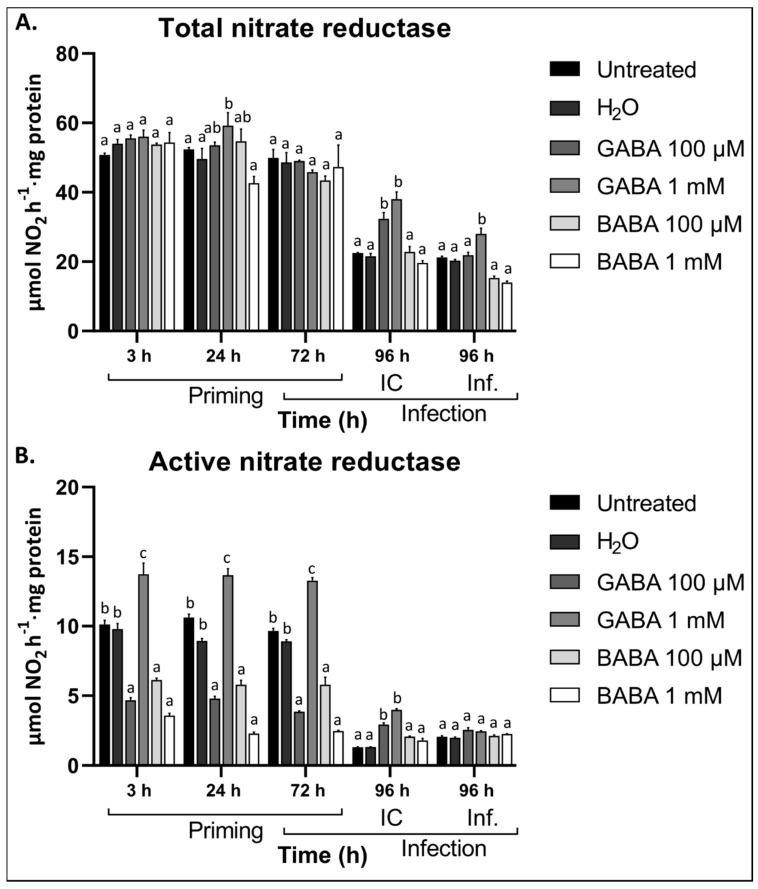
The effect of exogenous GABA treatment and *B. cinerea* infection on the activities of total and active nitrate reductase enzymes in the leaves of *Arabidopsis* plants. The activity of (**A**) total and (**B**) active nitrate reductase in response to treatment with 100 µM or 1 mM exogenous GABA or BABA followed by infection with *B. cinerea*. Untreated and H_2_O-treated plants were used a control. Bars represent the mean ± SE of 3 biological replicates. Statistical significance is indicated with different letters (a, b and c) (*p* < 0.05) within the same timepoint and is based on two-way ANOVA and Tukey’s multiple comparison test and adjusted *p* values. Experiment was repeated three times with consistent results. IC: infection control. Inf: Infection.

The source leaves of plants were considered for NR activity. Total NR activity represents both the active and inactive forms, whereas active NR represents the unphosphorylated (actual) NR activity. Our data indicate that GABA differentially regulates NR activity depending on the concentration. Contrary to 100 µM exogenous GABA, 1 mM stimulated the total NR activity 24 h after treatment (Figure 5A). The active NR, on the other hand, showed distinct differences between GABA treatments at 100 µM and 1 mM (Figure 5B). At 1 mM, the active NR activity was significantly induced compared to the controls, whereas at 100 µM GABA the activity was reduced. This effect was observed at all the timepoints considered after treatment. BABA treatments resulted in a similar effect to GABA at 100 µM, inhibiting the active NR activity (Figure 5B). Leaf defoliation resulted in a reduction in both the total and the active NR activity, irrespective of the treatment (difference between 72 and 96 h IC). Interestingly, after leaf defoliation, the total and active NR activity was significantly higher in plants pre-treated with GABA at both 100 µM and 1 mM (Figure 5A,B). After infection, however, only plants treated with 1 mM showed higher levels of total NR activity compared to the controls. Pre-treatment with BABA did not significantly affect the NR activity after infection. A comparison of the NR activity and the leaf GABA levels suggests that 1 mM exogenous GABA is able to promote nitrogen utilization and endogenous GABA accumulation, whereas 100 µM did not initiate such a response. 

### 3.6. GABA Treatment Induces Soluble Sugar Accumulation in Response to B. cinerea Infection

It is surprising that there are few data available on the interplay between GABA and soluble sugar metabolism and signalling. To establish whether exogenous GABA affects soluble sugar content after treatment and *B. cinerea* infection, we analysed the levels of glucose (Glc), fructose (Fru), and sucrose (Suc) in the source leaves after leaf spraying plants with 100 µM and 1 mM GABA and BABA, in comparison to untreated and H_2_O controls.

**Figure 6:**
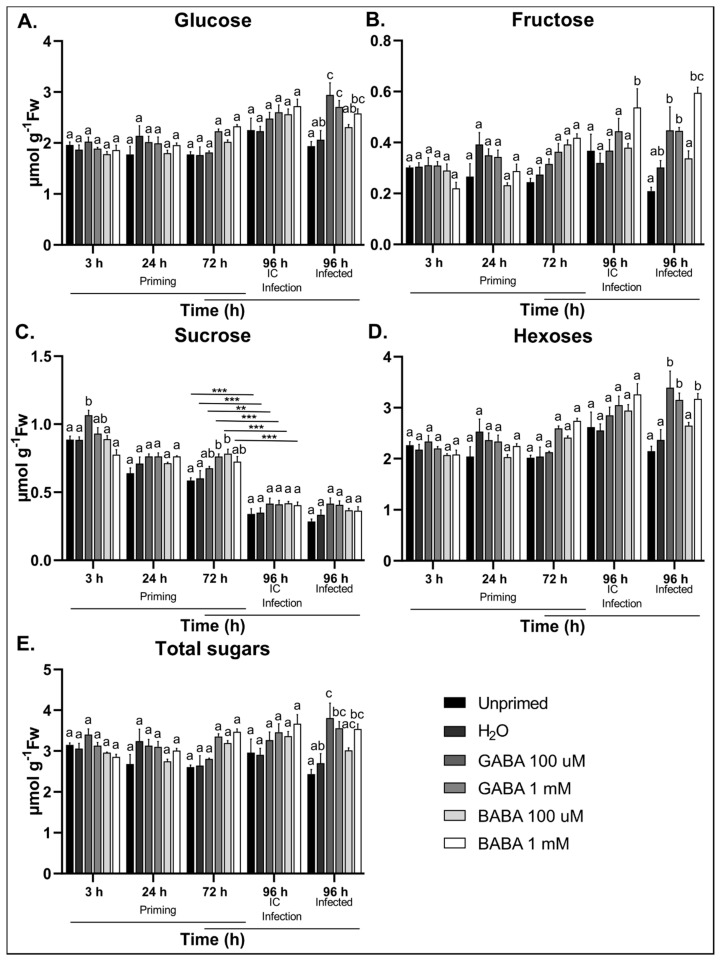
Analysis of soluble sugar content after treatment with GABA or BABA followed by infection with *B. cinerea*. (**A**) Glucose, (**B**) fructose, and (**C**) sucrose levels in response to GABA and BABA treatment at 100 µM and 1 mM followed by *B. cinerea* infection. (**D**) Total hexoses (glucose + fructose) and (**E**) total sugars (glucose + fructose + sucrose). Bars are the mean ± SE of 6 biological repeats. Statistical significance is indicated by different letters (a, b and c) within the same timepoint (*p* < 0.05) and with an asterisk between different timepoints (** *p* < 0.01; *** *p* < 0.001) based on two-way ANOVA followed by Tukey’s multiple comparison test and adjusted *p* values. Data is representative of one experiment from 3 independent repeats with consistent results.

For hexoses and total sugars, no significant differences were detected between the different treatments at the pre-infection stages (Figure 6A,B,D,E). On the other hand, Suc levels significantly increased at 3 and 72 h after treatment with 100 µM and 1 mM GABA, respectively (Figure 6C). Plants treated with BABA also showed Suc accumulation 72 h after treatment (Figure 6C). Leaf detachment was associated with a significant decrease in Suc levels independent of the treatment (Figure S5A). After *B. cinerea* infection, plants pre-treated with GABA at both 100 µM and 1 mM showed significantly higher levels of Glc, Fru, hexoses, and total sugars (Figure 6A,B,D,E). Plants pre-treated with BABA also showed significant accumulation of Glc, Fru, hexoses, and total sugars after *B. cinerea* infection compared to the control (Figure 6A,B,D,E). Only BABA treatment at 1 mM significantly increased the ratio of hexoses to Suc in response to *B. cinerea* infection (Figure S5B). Thus, GABA treatments did not significantly affect Glc or Fru levels directly but did induce time-dependent Suc accumulation compared to controls. On the other hand, GABA treatments caused a significant accumulation of hexoses after infection with *B. cinerea* in comparison with the controls. The increase in soluble sugars in response to GABA treatment correlates with increased levels of GABA and total NR activity after *B. cinerea* infection, suggesting that GABA promotes metabolic activity during infection.

## 4. Discussion

The urgent need to use natural and less toxic approaches to control pathogens in agriculture has increased the interest to prime plants with compounds of natural origin [67,68]. One of the best-studied priming compounds, BABA, is well characterized to induce resistance against several (a)biotic stresses [69,70,71]. Far less is known about the priming ability and mode of action of GABA, a structural isomer of BABA.

Higher concentrations of exogenous GABA (1–10 mM) are able to induce resistance in tomato leaf disks and fruits against *B. cinerea* [30,72]. Our data illustrate for the first time that lower concentrations of exogenous GABA (100 µM) are sufficient to prime *Arabidopsis* plants for extended periods (72 h) against the necrotrophic fungus *B. cinerea* (Figure 1). Identical concentrations of BABA and GABA generated similar protection against *B. cinerea* infection (Figure 1A), although GABA was more effective than BABA at 100 µM, focusing on the most severe lesion category. Realistically, during foliar spraying, only a small percentage (e.g., 5–10%) will be able to penetrate the cuticula and cell wall to reach membrane receptors and/or transporters [73]. Still, 5–10 µM would be available for signalling, which is well in line with the affinity of animal GABA and Glu receptors [74,75,76]. In any case, the fact that such low GABA concentrations can effectively prime resistance suggests that GABA signalling rather than metabolic effects are at play. One possible physiological explanation is that stressed cells accumulating GABA in the cytosol as a consequence of abiotic or biotic stress can release GABA at lower concentrations into the apoplast. This may occur by the fact that cytosolic GABA, above a certain threshold, induces its own export by aluminium-activated malate transporters (ALMTs) [71,72,73]. Certain pathogens were proposed to highjack such GABA exporters, leading to a very high concentration of GABA in the apoplast of tomato [14]. Alternatively, cellular damage or leakage can also release GABA in the apoplast, acting as a DAMP and functioning as a “danger” signal when perceived by neighbouring cells. Similarly, as proposed for extracellular sugar signalling, the sensitivity of the neighbouring cells may be determined by the history and the energetic status of these cells, with N- and C-starved cells being less receptive to “danger” signalling and more receptive to “food” signalling [77,78].

As a putative signalling molecule in plants, it is no surprise that GABA is closely associated with cellular ROS dynamics. Exogenous GABA has been shown to differentially control the expression of an NADPH-oxidase gene during salt stress in the roots of *Caragana intermedia* [47]. With ROS being a crucial component of the infection strategy of *B. cinerea*, the timing and distribution of plant ROS production in response to *B. cinerea* is crucial for plant resistance. Our data also showed a concentration-dependent regulation of NADPH-oxidases (Figure 2). It is interesting though that in animals, GABA-mediated pain repression pathways during Parkinson’s disease are negatively regulated by NADPH-oxidases [79]. Alternatively, it has been shown that 14-3-3 proteins act as positive regulators of NADPH-oxidases [80] and because GABA also regulates 14-3-3 proteins, it can be hypothesized that GABA functions through 14-3-3 proteins to regulate NADPH-oxidases upon elicitor perception [15]. The timing of such regulatory events seems crucial, since an initial ROS burst can be effective to induce tolerance [81,82], whereas overactivation of NADPH-oxidases at the necrotrophic stage of the pathogen increases susceptibility [49,63,83]. Data on the role of NADPH-oxidases during *B. cinerea* infection often appear contradictory, but this may be caused by differential time windows used among researchers. Nevertheless, lowering the activity of NADPH-oxidases at the later infection stages might favour the plant to prevent the development of necrosis [49,84].

In contrast to BABA, treatment with GABA did not significantly affect the levels of H_2_O_2_ in the leaves, however, plants pre-treated with GABA did not accumulate significant amounts of H_2_O_2_ after infection with *B*. *cinerea* (Figure 3A,B). This supports previous findings illustrating that GABA reduced H_2_O_2_ levels in response to several stresses [44,45,46]. Reduced H_2_O_2_ content is likely due to direct activation of CAT after the priming treatment, and higher CAT and GPX activities after infection with *B. cinerea* (Figure 3C–E). Our data suggest that GABA primes the activities of ROS scavenging enzymes for a faster/more severe response after infection with *B. cinerea*, whereas BABA directly activated APX and GPX without the presence of the pathogen. Previous data indicated that a combination of GABA and (a)biotic stress is required to induce ROS scavenging enzymes [45,46]. Lowered activities of NADPH-oxidases together with increased ROS scavenging enzymes suggest that GABA promotes more reduced conditions, slowing down the infection process.

It has been demonstrated that 10 mM exogenous D6-GABA is rapidly taken up and metabolized into D4-succinic acid in leaves, while root feeding resulted in a fast distribution of D6-GABA over the whole plant [85]. Thus, transporters facilitating diffusion of GABA alongside a concentration gradient should be available within the plasma membrane. Candidate GABA importers include AtGAT1 and homologs of TaALMT [86,87]. It was demonstrated that TaALMT can both export and import GABA, with import being observed at an extracellular GABA concentration above 1 mM [86]. Considering the 1 mM sprays, time-dependent endogenous GABA levels are in line with a process of cellular uptake, reaching a maximum, and decreasing afterwards because of being metabolised to succinate (Figure 4A). Such endogenous GABA dynamics were not observed in the case of the 100 µM treatments, suggesting that uptake may not have been in play, pointing at GABA-mediated extracellular signalling processes instead. It is thus possible that extracellular GABA signalling and metabolism (uptake and linking to the TCA cycle) can function completely independently of one another at low enough concentrations. After infection, primed GABA plants clearly show increased GABA levels, probably through the stimulation of endogenous GABA synthesis and the further establishment of GABA waves at the tissue and whole plant level [22], perhaps similar to the recently established Glu/Ca^2+^ systemic waves involved in plant defence signalling [88,89].

With the notion that GABA signalling and metabolism can be separated at specific concentrations, we found that exogenous GABA at 1 mM promoted both the total and active NR activity whereas 100 µM GABA inhibited active NR activity (Figure 5). The increase in NR activity at 1 mM treatment correlated with higher leaf GABA content, suggesting that high endogenous GABA might be responsible for the increased NR activity. It is therefore possible that extracellular GABA at a low concentration might emulate a stress condition as proposed previously [47]. As such, GABA can be perceived at the plasma membrane, inducing Ca^2+^, which is known to activate several defence responses [90,91], but also inhibit NR activity [92]. On the contrary, at 1 mM, GABA is likely imported to fuel the GABA shunt, activating its own synthesis to fuel the TCA cycle. This requires more C and N to support metabolic processes, necessitating the stimulating NR enzyme activation. Alternatively, it is also known that GABA can regulate some 14-3-3 proteins [15], which are key regulators of phosphorylated NR activity [40]. It is interesting that at both concentrations, total and active NR activity increased after leaf defoliation (Figure 5), suggesting that GABA at lower concentrations is still sufficient to promote metabolism during subsequent stresses. This also illustrates a potential link between endogenous GABA content and the activity of NR, suggesting that high GABA levels might emulate high metabolic flux requiring an additional supply of nitrogen.

Connections between GABA and sugar metabolism/signalling are poorly established. Suc signalling events are typically associated with temporal fluctuations in Suc levels, which often involves the production of secondary metabolites with antifungal activity [93,94]. As such, temporal Suc fluctuations observed in our data can lead to an early boost in defence arsenal after subsequent infection. In response to infection, however, hexoses significantly accumulated in plants treated with either GABA or BABA. Exactly how GABA induces the accumulation of soluble sugars is a subject of further investigation. However, it might be attributed to a GABA-mediated retainment of photosynthetic capabilities as reported for the abiotic stress context [43]. It is also possible that increased soluble sugars occur as a consequence of altered starch/soluble sugar balances [95]. The fact that Glc and Fru levels increased after infection supports the notion that GABA favours a pro-survival strategy, stimulating normal metabolic activities to fuel pathways, such as the GABA shunt and the TCA cycle, which are key components during plant stress responses [22,30]. Moreover, Glc also serves as the precursor for many antioxidants like ascorbic acid, which is a crucial scavenger of ROS during necrotrophic pathogen infection [96]. No clear link has been established between GABA signalling and SnRK1, but it can be hypothesized that lower levels of extracellular GABA, perceived as “danger”, may stimulate SnRK1, while high intracellular GABA levels may have the opposite effect. These are exciting new avenues in which 14-3-3 proteins may be involved, since GABA affects 14-3-3 proteins, which are also a key component in the regulation of SnRK1 target proteins [40].

## 5. Conclusions

Our data support GABA as an effective priming agent against *B. cinerea* at low concentrations. Several responses can be differentially regulated based on the concentration of GABA, suggesting GABA priming can be performed without inflicting major metabolic pathways. It will be interesting to repeat our experiments on plants lacking an array of GABA membrane transporters to better discriminate between extracellular and intracellular signalling events. In general, GABA promoted the detoxification of ROS through ROS scavenging enzymes, and increased GABA metabolism and sugar accumulation after infection with *B. cinerea*. This indicates that increasing GABA content signals/promotes metabolic processes and strategies that prevent toxification and cell death. Our data support a role of low GABA concentrations to be perceived extracellularly as stressors and higher concentrations to be taken up and act as a signal of favourable (nutrient availability) conditions. Knowing that mammalian GABA receptor antagonists also abolish the effect exerted by GABA in plants, it is possible that shared mechanisms are into play. As such, it is possible that GABA signalling can be translated to distal tissues and organs through constant secretion/uptake of GABA between cells, as it is the case in animals.

## Supplementary Materials

Supplementary materials can be found at https://www.mdpi.com/2076-3921/9/12/1174/s1, Figure S1: Picture representing *B. cinerea* lesions 72 h after infecting *Arabidopsis* source leaves. Rosette leaves 5–7 were detached 72 h after treatment with either GABA or BABA and infected with 5 µL droplets of *B. cinerea* spores for 72 h, Figure S2: Scheme illustrating the experimental setup used in this study for priming, infection and sampling of material. Plant treatment at 0 h followed by ROS burst experiments at 24 h after treatment. Infection with occurred 72 h after treatment, and disease analysis 72 h after infection. Samples were harvested at 3 h, 24 h, 72 h and 96 h after treatment, Figure S3: (A) flg22-and (B) OGs-induced ROS burst in untreated and H_2_O treated plants. Untreated *Arabidopsis* or plants pre-treated with H_2_O containing 0.0001 % Tween-20 were treated with 100 nM flg22 or 0.2 mg/mL OGs 24 h later to assess the effect of H_2_O spraying on ROS burst. Values represent the mean of 8 biological replicates, Figure S4: Effect of GABA treatment on the ROS burst in leaf disks compared to OGs as positive control and H_2_O as negative control. Values represent the mean of 8 biological replicates, Figure S5: (A) Decrease in Suc content as a consequence of leaf defoliation after 24 h. Statistical significance is indicated by different letters (*p* < 0.05) within the same timepoint or treatment or with an asterisks (* *p* < 0.05; ** *p* < 0.005; *** *p* < 0.001) between different timepoints and is based on two-way ANOVA followed by Tukey’s multiple comparisons test. (B) Effect of GABA and BABA priming on the ratio of hexoses to sucrose. Statistical significance is indicated by different letters (* *p* < 0.05) within the same timepoint and is based on two-way ANOVA followed by Tukey’s multiple comparisons test.
