# Supplementary figures and images for: Priming with γ-Aminobutyric Acid against Botrytis cinerea Reshuffles Metabolism and Reactive Oxygen Species: Dissecting Signalling and Metabolism
Source: Antioxidants (Basel). 2020 Nov 25;9(12):1174. doi: 10.3390/antiox9121174 (PMC7759855; doi:10.3390/antiox9121174)

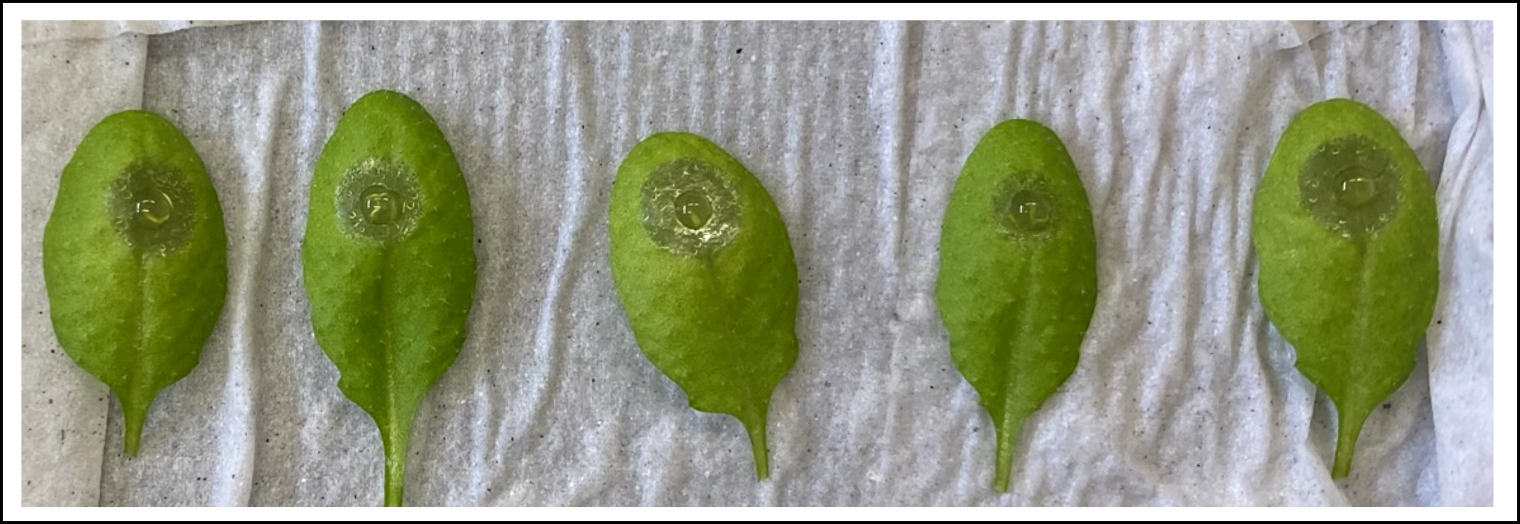

Supplement: Supplementary file 1 [file antioxidants-09-01174-s001.zip › antioxidants-1022717-supp-final check/Figure S1.png]

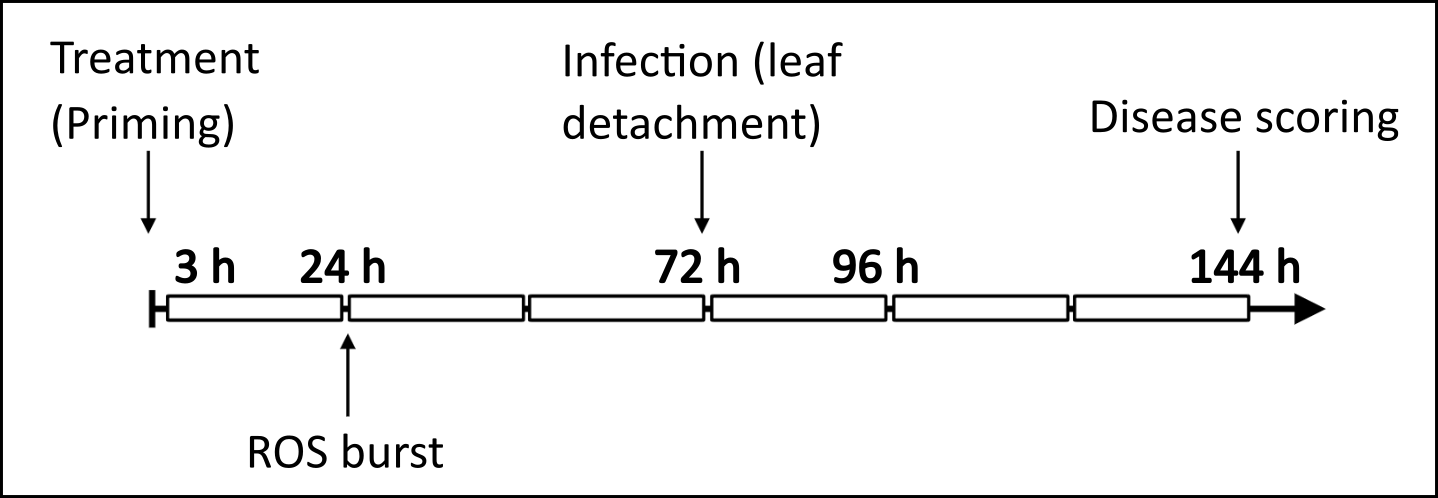

Supplement: Supplementary file 1 [file antioxidants-09-01174-s001.zip › antioxidants-1022717-supp-final check/Figure S2.png]

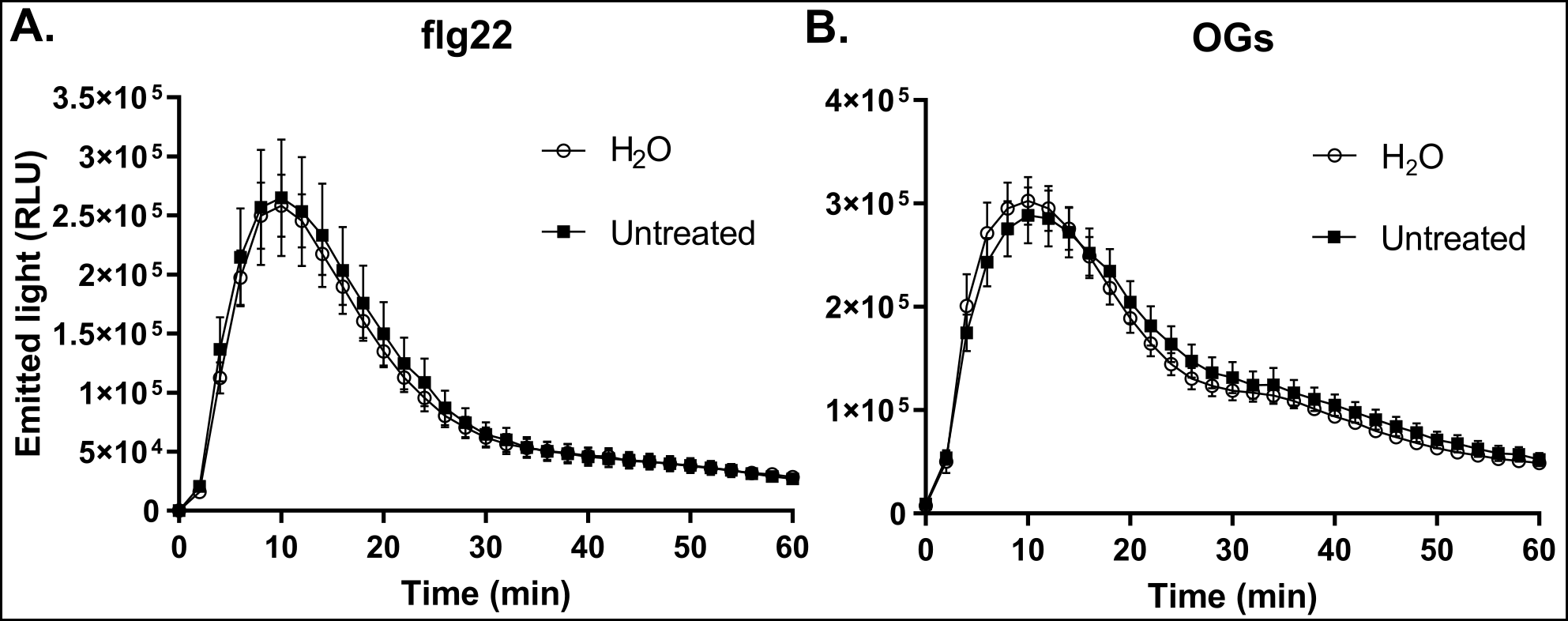

Supplement: Supplementary file 1 [file antioxidants-09-01174-s001.zip › antioxidants-1022717-supp-final check/Figure S3.png]

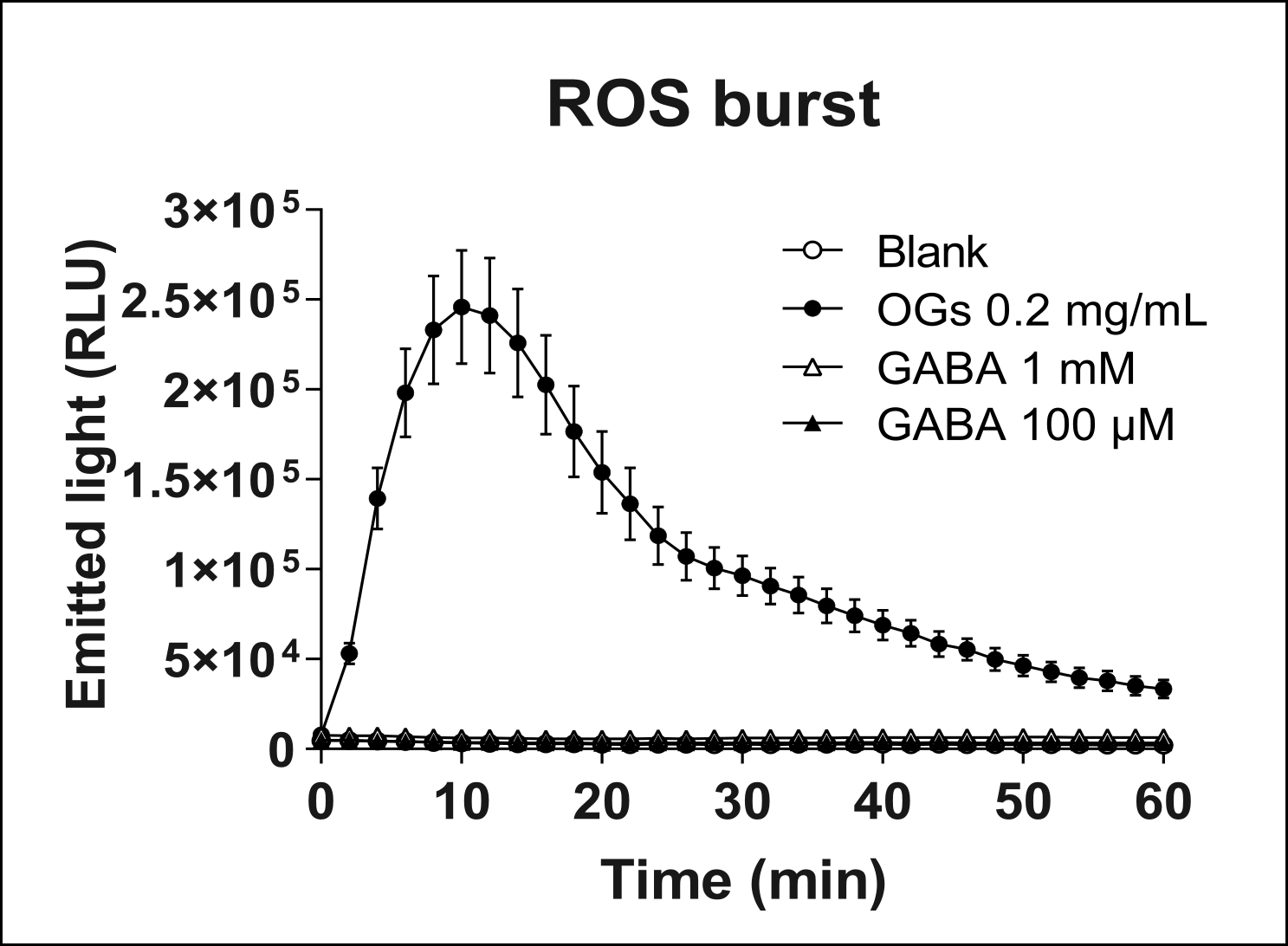

Supplement: Supplementary file 1 [file antioxidants-09-01174-s001.zip › antioxidants-1022717-supp-final check/Figure S4.png]

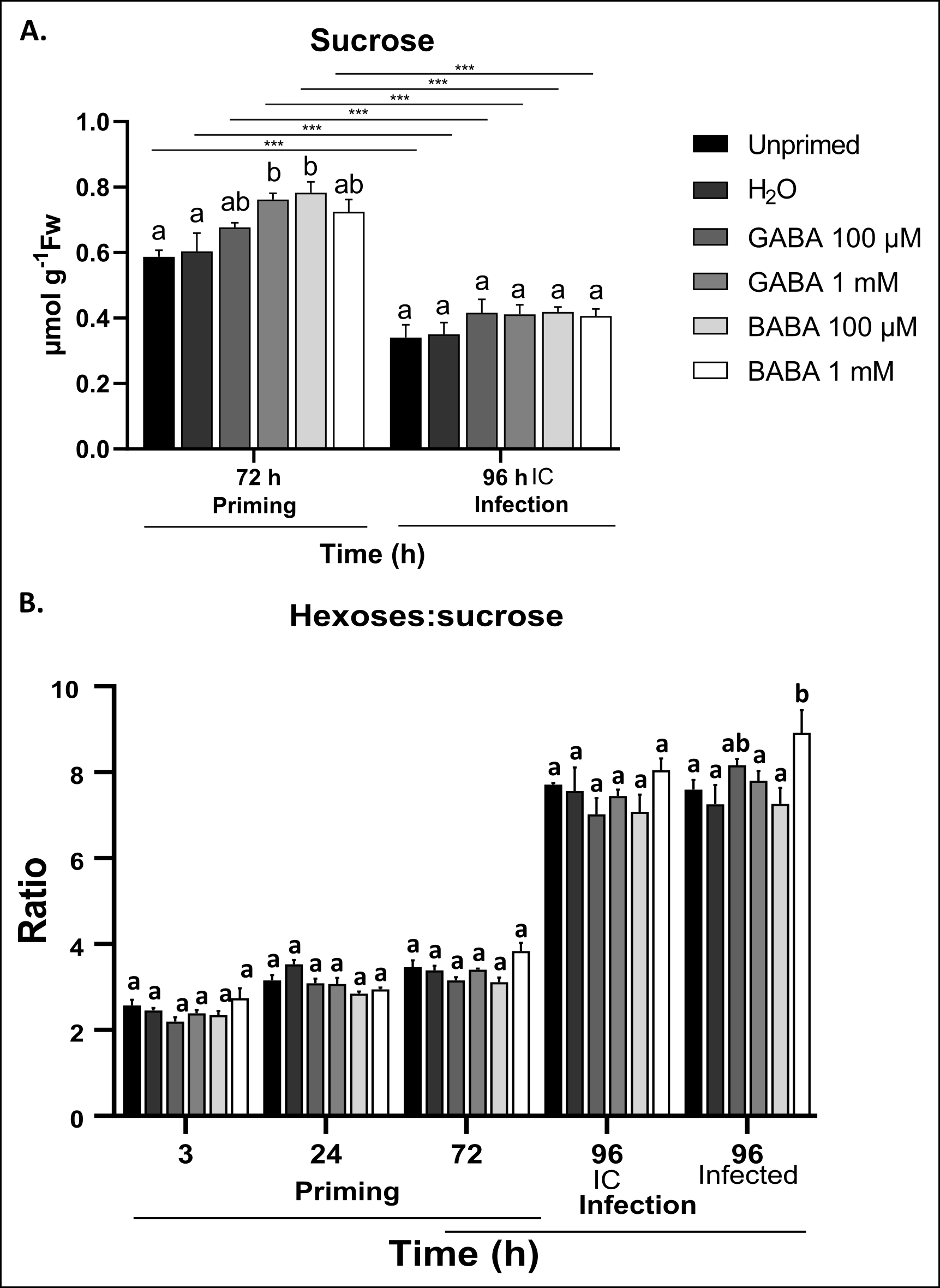

Supplement: Supplementary file 1 [file antioxidants-09-01174-s001.zip › antioxidants-1022717-supp-final check/Figure S5.png]
